# Diseases of the Stomach

**Published:** 1904-10-29

**Authors:** 


### Progress in Medicine and Surgery.

### DISEASES OF THE STOMACH.

(*Concluded from page* 71.)

Gastric Ulcer.—The frequency of this condition is shown by the fact that out of 311 cases of disease of the stomach, published by Calwell,[10] 193 were gastric ulcer. In women none of these began before the age of 13 years, while in men none occurred before 25. The greatest number of cases in women were between 17 and 19 years, and both in women and men very few cases began after 25 years. The early cases (developmental) were confined to women and corresponded to the cases of chlorosis in age incidence. Return of pain on getting the patient up he attributes to adhesions or kinking of the first part of the duodenum. The best treatment he considers to be rest in bed and milk diet; Dempsey[11] recommends a powder of magnesia, bismuth and soda, and later on oxide of silver. Wagner,[12] however, has published 60 cases with hæmatemesis and only one death treated on Lenhartz's plan, which consists in giving a concentrated albuminous diet with a view of strengthening the patient and diminishing the hyperchlorhydria which causes the ulceration. For the first few days 30 grains of bismuth subnitrate are given frequently, and after a week, Blaud's pill. On the day of the hæmorrhage 200 to 300 cc. of iced milk (seven to ten ounces) in teaspoonful doses with one, two, or three whipped eggs are given. The amount is increased till at the end of a week the patient gets 800 cc. (28 ounces) and six or eight eggs. From the third to the sixth day raw scraped meat is also given. In three or four weeks full mixed diet is given. The pain is generally gone in a week, and the treatment can usually be stopped in two months. Out of 60 patients subsequent reports were received from 25, of whom 18 were well and able to work, and only two had had recurrence of hæmorrhage. It is important to distinguish gastric ulcer from another condition common in young chlorotic girls, namely gastric hyperæsthesia. Soltau Fenwick[13] divides this condition into four stages, (1) there is fulness and discomfort after each meal, radiating burning pains in the chest, flatulence, nausea and constipation. (2) Genuine pain, directly after meals and worst when solid food has been taken. (3) Vomiting appears, increasing in severity, till it occurs after every meal. It does not relieve the pain, and there is no loss of weight or appetite. (4) Nutrition fails, vomiting follows too rapidly after eating for pain to be present. The diagnosis from gastric ulcer lies in the presence of cutaneous hyperæsthesia (two-thirds of the cases), general not localised tenderness, more frequent vomiting which is immediate and does not relieve the pain, and the failure of rest in bed and milk diet to improve the condition. The treatment consists in soft, rather than liquid foods, and a return to ordinary diet as soon as possible. The anæmia and constipation should be treated, but gastric sedatives and enemata should not be employed. Duplant[14] has called attention to the adhesions which may follow on gastric ulcer, and the ease with which the symptoms produced may simulate carcinoma. If near the pylorus (a rarer form) stenosis is usual and jaundice may occur. A tumour simulating cancer may be felt and the diagnosis must rest upon the history and the absence of secondary growths in the liver or glands. The treatment is gastro-enterostomy. If the adhesions are near the cardiac end of the stomach, hour-glass deformity may result; but not pyloric stenosis or dilatation. The symptoms usually occur after many years, but may come on soon after the ulcer, a localised abscess is then present. The pain differs from that of ulcer in radiating to the shoulder or left breast, and not extending below the umbilicus. Exacerbations with increase in the size of the tumour are due to peritonitis. The tumour, which may be continuous with the left lobe of the liver, is distinguished from a secondary carcinoma by its strict limitation to the edge of that organ, and from gastric carcinoma by its superficial position and the fact that it causes no obstruction of the cardiac orifice. The treatment, in cases which resist the ordinary medical measures for ulcer, is to divide the adhesions and perform gastro-enterostomy.

**Malignant Disease.**—Three hundred and six cases have been analysed by Perry and Shaw[15] from the post-mortem records of Guy's Hospital, between the years 1826 and 1900. The condition was found in 1·4 per cent. of all autopsies. Eighteen patients of both sexes died at the age of 45, the largest number for any one year, and more than one-third died between 40 and 50. Of 300 cases 218 were males and 82 females. The average duration of the disease was eight and three-quarter months, but in two cases carcinoma may have existed for four years. In all but two cases there was definite pyloric obstruction, but dysphagia was only noted in 10 cases. Hæmatemesis occurred in rather more than one-sixth of the cases, of which six were fatal, in four others fatal hæmorrhage into the stomach occurred. There were 32 cases of acute peritonitis, 12 after operation, 13 after perforation, seven from softening of secondary growths. The absence of symptoms of peritonitis often seen was attributed to the already serious condition of the patients, the absence of HCl from the stomach contents, and the presence of ascitic fluid diluting the extravasated matter. Arncill[16] shows that laboratory tests alone are of doubtful value in the diagnosis of carcinoma, the persistent absence of HCl being more constant in pernicious anæmia than in malignant disease. Mouisset and Tolot[17] have shown that while a fall in number of the red blood cells is merely due to the onset of cachexia, a fall in the hæmoglobin value is a useful sign in the diagnosis from cirrhosis of the liver, gastric ulcer, or catarrh and pernicious anæmia. Leucocytosis is also coincident with the onset of cachexia. The differential count has no diagnostic value.

**Chronic Disorders.**—From the point of view of diagnosis chronic digestive disturbances are divided by Herschell[18] into those requiring an examination of the stomach contents and those in which a diagnosis can be made by ordinary clinical observations. Among the latter he places slight temporary derangements due to obvious errors of diet, slighter cases of nervous dyspepsia, simple hyperchlorhydria in young men, and dyspepsia in chlorotic girls. All these can be diagnosed by the history and symptoms. In suspected cases of ulcer and in digestive disturbances due to organic disease of the heart the passage of a stomach-tube is positively contra-indicated, as it can add little to our knowledge and may do great damage. Where, however, there are signs of gastritis, where the trouble has resisted treatment for a long time, and where there is loss of flesh, a sudden commencement during middle life, or an evident tendency to get worse, further methods of ascertaining the condition of the digestive functions are necessary. The important points which must be determined are: The presence or absence of gastritis, the motor power of the stomach, the period during which the gastric juice is secreted and its character, the presence or absence of gastroptosis or dilatation, the amount of HCl deficit, if any, and the presence of the rennet ferment. The routine examination will be: After a midday meal of bread and meat, the stomach is emptied at 7 P.M. If there is no residue the motor functions are normal; if there is a residue, the stomach should be washed out and a dinner of meat and bread given. The next morning the stomach contents are again extracted, and if the motor insufficiency is sufficiently pronounced food residues will again be found, but a few cubic centimetres of greenish, slightly acid fluid is not abnormal. More than 20 c.c. of fluid, with a considerable amount of free HCl, shows hypersecretion. The stomach may be inflated before the tube is removed, and its size and position determined. It is then washed out and Ewald's test breakfast given. One hour later the total acidity, free HCl and peptic power of the stomach contents are to be determined. Hyperchlorhydria is shown by bad digestion of bread, high total acidity, and much free HCl. Chronic gastritis by tolerable digestion of bread with low acidity, little free HCl, and the presence of excess of mucus. Malignant disease, chronic acholia, and the terminal stage of chronic gastritis, all give rise to bad bread digestion and failure of the filtered contents to digest albumen or coagulate milk on the addition of calcium chloride. These conditions must be differentiated by the history and course of the disease. Progressive stenosis of the pylorus and myasthenia may be distinguished by giving the patient fasting 300 c.c. of water to drink, followed in an hour by 100 c.c. 1 per cent. glucose solution. The sugar is then estimated in a few ounces withdrawn by the tube. A high percentage will show that most of the 300 c.c. have left the stomach, and that consequently stenosis and hypertrophy are probably present. Malignant disease in its earlier stages may be determined by washing out the stomach and then giving a meal of porridge made of sterilised oatmeal. If, after such a meal, lactic acid is found in the stomach it shows that the motor power is deficient, and that much mucus has been left clinging to the stomach wall after the washing. This mucus contains the lactic acid bacilli, and thus lactic acid is formed from the porridge. Malignant disease is practically the only condition in which this can take place. Hayes[19] has noted the occasional occurrence of tetanic spasms in connection with various chronic digestive disturbances. Chronic and exhausting vomiting or diarrhœa are often associated with it. The condition may closely simulate strychnine poisoning, or tetanus, and the writer believes that many cases of idiopathic tetanus which have recovered or been cured by tetanus antitoxin are in reality of this nature.

[10] Lancet. April 23, 1904, p. 1125. [11] Ibid. [12] Edin. Med. Journ., March, 1904, p. 270. [13] Clin. Jour., Feb. 24, 1904, p. 296. [14] Practitioner, May, 1904, p. 745. [15] Guy's Hospital Reports, vol. lviii., p. 121. [16] Am. Med., Jan. 16, 1904, p. 93. [17] Practitioner, May, 1904, p. 753. [18] Clin. Jour., April 6, 1904, p. 385. [19] Dublin Jour., April, 1904, p. 272.

